# Mild Oxidative Stress Induced by Sodium Arsenite Reduces Lipocalin-2 Expression Levels in Cortical Glial Cells

**DOI:** 10.3390/ijms242115864

**Published:** 2023-11-01

**Authors:** Ye-Jin Cho, So-Hyun Park, Kwon-Yul Ryu

**Affiliations:** Department of Life Science, University of Seoul, Seoul 02504, Republic of Korea; fifi1022@naver.com (Y.-J.C.); sohyun1231@naver.com (S.-H.P.)

**Keywords:** astrocyte, lipocalin-2, oxidative stress, glial cells, sodium arsenite

## Abstract

Astrocytes and microglia, the most abundant glial cells in the central nervous system, are involved in maintaining homeostasis in the brain microenvironment and in the progression of various neurological disorders. Lipocalin-2 (LCN2) is a small secretory protein that can be transcriptionally upregulated via nuclear factor kappa B (NF-κB) signaling. It is synthesized and secreted by glial cells, resulting in either the restoration of damaged neural tissues or the induction of neuronal apoptosis in a context-dependent manner. It has recently been reported that when glial cells are under lipopolysaccharide-induced inflammatory stress, either reduced production or accelerated degradation of LCN2 can alleviate neurotoxicity. However, the regulatory mechanisms of LCN2 in glial cells are not yet fully understood. In this study, we used primary astroglial-enriched cells which produce LCN2 and found that the production of LCN2 could be reduced by sodium arsenite treatment. Surprisingly, the reduced LCN2 production was not due to the suppression of NF-κB signaling. Mild oxidative stress induced by sodium arsenite treatment activated antioxidant responses and downregulated *Lcn2* expression without reducing the viability of astroglial-enriched cells. Intriguingly, reduced LCN2 production could not be achieved by simple activation of the nuclear factor erythroid-2-related factor 2 (Nrf2)–Kelch-like ECH-associated protein 1 (Keap1) pathway in astroglial-enriched cells. Thus, it appears that mild oxidative stress, occurring in an Nrf2-independent manner, is required for the downregulation of *Lcn2* expression. Taken together, our findings provide new insights into the regulatory mechanisms of LCN2 and suggest that mild oxidative stress may alter LCN2 homeostasis, even under neuroinflammatory conditions.

## 1. Introduction

In the central nervous system, astrocytes and microglia perform diverse physiological functions, including the control of extracellular ion concentration, nutrient provision, and the regulation of blood flow by interacting with endothelial cells in the blood–brain barrier [1,2]. Moreover, they are involved in several neurological disorders through reactive astrogliosis [3]. The conventional classification of reactive astrocytes as A1 or A2 is based on their molecular and cellular characteristics [4]. According to a previous study, A1 astrocytes are induced by the activation of nuclear factor kappa B (NF-κB) signaling, while A2 astrocytes are induced by the activation of Janus kinase (JAK)/signal transducer and activator of transcription 3 (STAT3) signaling [5,6]. A1 and A2 astrocytes show high expression levels of pro-inflammatory and anti-inflammatory cytokines, respectively. A1 astrocytes are considered to be neurotoxic as they secrete neurotoxins, such as lipocalin-2 (LCN2), and A2 astrocytes are neuroprotective as they secrete neurotrophic factors, such as brain-derived neurotrophic factor (BDNF) or vascular endothelial growth factor (VEGF) [7,8,9]. In neuroinflammatory diseases, several cytokines derived from other cells, such as microglia, induce massive transcriptome alterations in quiescent astrocytes, resulting in a dominant population of A1 astrocytes and the secretion of neurotoxic molecules, including LCN2 [10]. However, it is widely accepted that the characteristics of reactive astrocytes should be considered in a context-dependent manner, rather than the traditional dichotomous concept that A1 astrocytes are neurotoxic and A2 astrocytes are neuroprotective [11,12].

LCN2, also known as neutrophil-associated gelatinase lipocalin or 24p3, has been identified as an acute-phase protein as its concentration dramatically increases in the blood under inflammatory stress conditions [13,14]. This secretory protein is conserved between mice and humans and it binds to several types of siderophores, which are strong iron utilizers found in some infectious bacteria and fungi, resulting in the sequestration of iron-utilizing molecules and inhibition of the growth of the bacteria and fungi. Therefore, increased LCN2 levels in plasma and several tissues, such as the kidney, lung, and brain, are often observed in pathological conditions [15,16,17,18]. *Lcn2* expression is regulated by several cell signaling pathways that are activated under inflammatory stress conditions, such as the NF-κB signaling and JAK/STAT3 signaling pathways [19]. Tumor necrosis factor alpha (TNF-α) and interferon gamma (IFN-γ) are required for the upregulation of *Lcn2* expression in various cell types [19,20]. Recently, several studies have found that LCN2 is produced and secreted by reactive astrocytes or activated microglia in the nervous system and is involved in the pathological progression of neuroinflammatory diseases or the restoration of nervous tissue after ischemic injury [21,22]. Of note, LCN2 secreted from reactive astrocytes can affect both damaged and healthy neurons [7]. 

Herein, we demonstrated that LCN2 production in primary astroglial-enriched cells can be significantly reduced under mild oxidative stress conditions induced by sodium arsenite treatment. Mild oxidative stress did not affect the viability of astroglial-enriched cells. Although activation of the nuclear factor erythroid-2-related factor 2 (Nrf2)–Kelch-like ECH-associated protein 1 (Keap1) pathway, the major antioxidant response pathway, has been suggested to suppress NF-κB signaling based on their molecular crosstalk, these two pathways may not interact or their interaction might be complex in astroglial-enriched cells [23]. Neither the activation of the Nrf2-Keap1 pathway nor the suppression of NF-κB signaling was the cause of reduced *Lcn2* expression under sodium arsenite treatment. Mild oxidative stress, induced by sodium arsenite treatment in an Nrf2-independent manner, seems to be responsible for the downregulation of *Lcn2* expression. Our current study suggests a novel strategy for reducing LCN2 levels to prevent its potentially neurotoxic effects without altering the NF-κB signaling pathway.

## 2. Results

### 2.1. Astroglial-Enriched Cells Are Resistant to Mild Oxidative Stress without Reduced Viability

**Figure 1:**
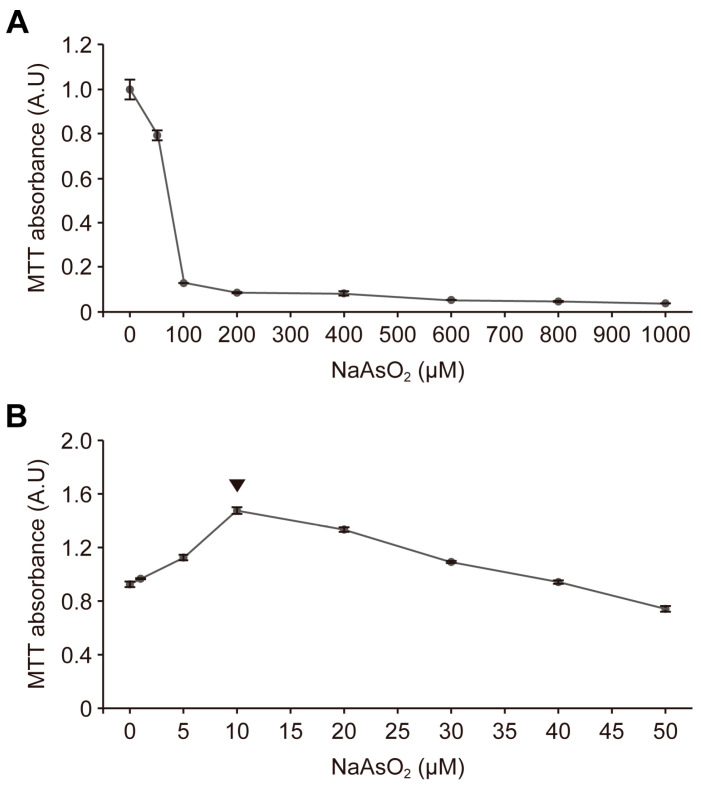
Astroglial-enriched cells survive under mild oxidative stress conditions without reduced viability. (**A**) astroglial-enriched cells were treated with sodium arsenite (NaAsO_2_) at the indicated concentrations up to 1000 μM for 1 d. The cells were then subjected to MTT assays. After incubation and elution with DMSO, the converted MTT level in each well was measured using a microplate reader. Data are expressed as MTT absorbance levels (arbitrary units, A.U; *n* = 3). (**B**) MTT assays were performed using astroglial-enriched cells, which were treated with NaAsO_2_ at the indicated concentrations up to 50 μM for 1 d. Data are expressed as described in (**A**) (*n* = 3). Arrowheads indicate the concentration of NaAsO_2_ that showed the highest MTT absorbance levels. All data are expressed as the means ± standard error of the mean (SEM) from the indicated number of samples.

When astroglial-enriched cells were exposed to sodium arsenite at concentrations of 100 μM or higher, their viability was markedly reduced (Figure 1A). According to previous studies, treatment with 10 μM sodium arsenite reduces the viability of primary neurons and mouse embryonic fibroblasts [24]. However, we found no reduction in the viability of astroglial-enriched cells when treated with the same concentration of sodium arsenite as used previously (10 μM), indicating that these cells may be more resistant to oxidative stress than other types of primary cells (Figure 1B). In fact, the LC50 of sodium arsenite was about 60 μM (Figure S1). Moreover, we observed that the MTT absorbance levels of astroglial-enriched cells did not markedly decrease until 50 µM sodium arsenite treatment, but rather increased with 10 μM sodium arsenite treatment (Figure 1B). Therefore, in this study, we defined the oxidative stress induced by sodium arsenite treatment of astroglial-enriched cells, without cytotoxicity, as “mild oxidative stress”.

### 2.2. Mild Oxidative Stress Activates the Nrf2–Keap1 Pathway and Reduces Lcn2 Expression Levels in Astroglial-Enriched Cells

**Figure 2:**
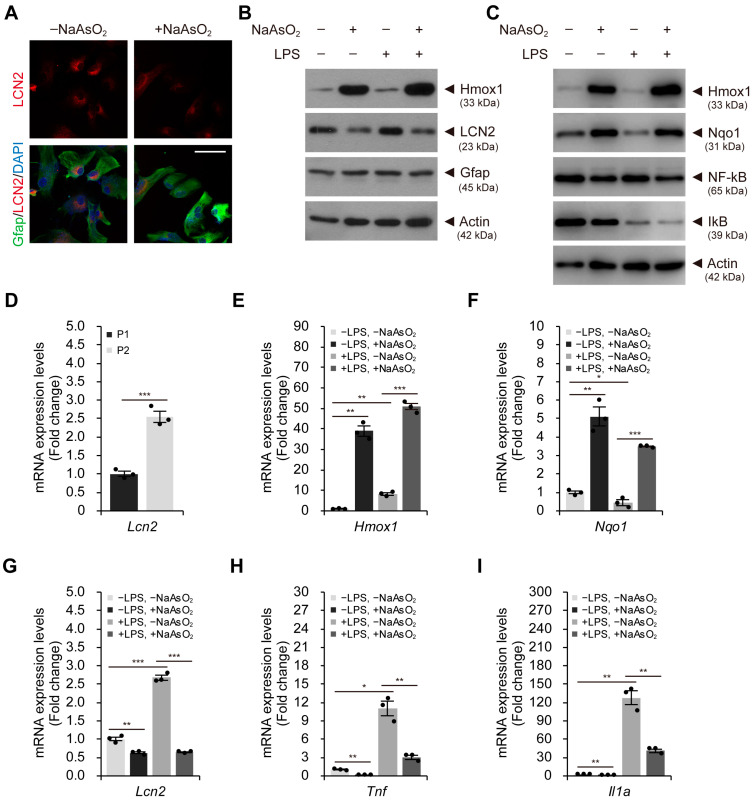
Mild oxidative stress activates the antioxidant response pathway and reduces LCN2 levels in astroglial-enriched cells. (**A**) immunofluorescence staining to detect intracellular LCN2 and Gfap in astroglial-enriched cells was performed with or without 10 μM NaAsO_2_ treatment. DNA was visualized with DAPI. (**B**) immunoblot detection of Hmox1, LCN2, and Gfap was performed using astroglial-enriched cells treated with 10 μM sodium arsenite (NaAsO_2_) or 100 ng/mL LPS for 1 d. Negative control cells were treated with an equal volume of PBS. (**C**) astroglial-enriched cells treated with 10 μM NaAsO_2_ and/or 100 ng/mL LPS for 1 d were subjected to immunoblot detection of Hmox1, Nqo1, NF-κB, and IκB. (**D**) the expression levels of *Lcn2* in astroglial-enriched cells at passage number 1 (P1) and 2 (P2) were determined by qRT-PCR, normalized against *Gapdh* levels, and expressed as the fold change relative to the control (P1; *n* = 3). (**E**–**I**) astroglial-enriched cells treated as described in (**C**) were subjected to total RNA isolation and qRT-PCR analysis. The expression levels of *Hmox1*, *Nqo1*, *Lcn2*, *Tnf*, and *Il1a* were determined by qRT-PCR, normalized against *Gapdh* levels, and expressed as the fold change relative to the control (−LPS, −NaAsO_2_; *n* = 3). For immunoblot analysis, β-actin was used as a loading control. Representative images of cells or immunoblots are shown (*n* = 3). qRT-PCR data are expressed as the means ± SEM from the indicated number of samples. Scale bar, 50 μm. * *p* < 0.05, ** *p* < 0.01; *** *p* < 0.001 between two groups as indicated by the horizontal bars.

We cultured astroglial-enriched cells without mechanistically removing microglia and oligodendrocytes until passage number two (P2). We found that glial fibrillary acidic protein (Gfap)-positive cells (astrocytes) were the dominant population (Figure 2A), although Gfap-negative cells (e.g., microglia) were also present under these conditions. Of note, we observed that prolonged exposure of astroglial-enriched cells to fetal bovine serum during the culture period induced their activation and subsequent production of LCN2, even without lipopolysaccharide (LPS)-induced inflammatory stress (Figure 2B,D). Although consistent expression of *Lcn2* in astroglial-enriched cells may not fully recapitulate normal physiological conditions, it is highly relevant to pathophysiological conditions and is suitable for investigating strategies to reduce LCN2 levels. 

Next, we investigated whether mild oxidative stress in astroglial-enriched cells activated the antioxidant response pathway. The levels of heme oxygenase 1 (Hmox1) and NAD(P)H quinone dehydrogenase 1 (Nqo1) in these cells increased under mild oxidative stress, suggesting that the Nrf2–Keap1 pathway was activated (Figure 2B,C and Figure S2A). Intriguingly, under the same conditions, intracellular LCN2 was reduced at the protein level, as confirmed by both immunoblot and immunofluorescence analyses (Figure 2A,B and Figure S2B). Moreover, reduced LCN2 levels and increased Hmox1 levels were also observed in astroglial-enriched cells treated with LPS under mild oxidative stress conditions (Figure 2B and Figure S2A,B). Thus, the effect of sodium arsenite seemed to override LPS-induced pro-inflammatory cytokine signaling, which can upregulate the expression of *Lcn2*. 

As expected, LPS treatment activated NF-κB signaling, as indicated by the reduced levels of inhibitory IκB (Figure 2C and Figure S2D). However, activation of the Nrf2-Keap1 pathway via sodium arsenite treatment did not appear to affect NF-κB signaling in astroglial-enriched cells (Figure 2C and Figure S2C). Furthermore, *Lcn2* expression levels increased significantly after passaging these cells to P2, even in the absence of NF-κB signaling activation due to LPS treatment (Figure 2D). Therefore, reduced LCN2 levels under mild oxidative stress do not seem to result from the suppression of NF-κB signaling (see Figure 2B). Consistent with the protein levels, *Hmox1* and *Nqo1* mRNA expression levels dramatically increased under mild oxidative stress conditions (Figure 2E,F). In addition, mild oxidative stress induced the downregulation of *Lcn2* expression in astroglial-enriched cells, regardless of LPS treatment conditions, which was consistent with the reduced LCN2 protein levels (Figure 2G). Interestingly, the increased expression levels of inflammatory markers due to LPS treatment were significantly decreased by sodium arsenite treatment (Figure 2H,I). Therefore, it is plausible that sodium arsenite may inhibit the transcription process of NF-κB target genes, either directly or indirectly, although it may not directly affect NF-κB signaling itself.

**Figure 3:**
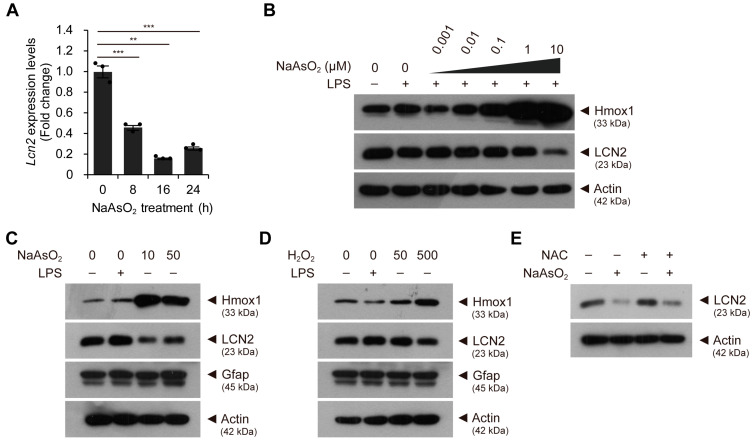
*Lcn2* expression levels are reduced by mild oxidative stress in a time-dependent manner. (**A**) astroglial-enriched cells were treated with 10 μM sodium arsenite (NaAsO_2_) for 0 to 24 h. *Lcn2* expression levels were determined by qRT-PCR, normalized against *Gapdh* levels, and expressed as the fold change relative to the control (0 h; *n* = 3). (**B**) immunoblot detection of Hmox1 and LCN2 was performed using control astroglial-enriched cells (without LPS or NaAsO_2_ treatment) and cells treated with 100 ng/mL LPS and various concentrations of NaAsO_2_ up to 10 μM for 1 d. (**C**) astroglial-enriched cells were treated with 100 ng/mL LPS or with 10 or 50 μM NaAsO_2_ for 1 d. The cells were then subjected to immunoblot detection of LCN2, Hmox1, and Gfap. (**D**) immunoblot analysis was performed, as described in (C), using astroglial-enriched cells treated with 100 ng/mL LPS or with 50 or 500 μM hydrogen peroxide (H_2_O_2_) for 1 d. (**E**) immunoblot detection of LCN2 in astroglial-enriched cells treated with 10 mM N-acetyl-L-cysteine (NAC) and/or 10 μM NaAsO_2_ for 1 d. To investigate the effect of NAC, 10 mM NAC was added 2 h before NaAsO_2_ treatment. For immunoblot analysis, β-actin was used as a loading control. Representative images of immunoblots are shown (*n* = 2). qRT-PCR data are expressed as the means ± SEM from the indicated number of samples. ** *p* < 0.01; *** *p* < 0.001 between two groups as indicated by the horizontal bars.

To further evaluate the effect of mild oxidative stress on astroglial-enriched cells, we treated the cells with sodium arsenite for different times or at different concentrations. We found that the expression levels of *Lcn2* decreased gradually with sodium arsenite treatment in a time-dependent manner for up to 24 h (Figure 3A). Furthermore, we observed that treatment with sodium arsenite at concentrations less than 10 µM did not sufficiently reduce the levels of LCN2 (Figure 3B and Figure S3A,B). Reduced levels of LCN2 were observed when the cells were treated with 50 µM sodium arsenite, although the effect was less than the effect of treatment at 10 µM (Figure 3C and Figure S4A,B). We also determined whether the reduction in LCN2 levels by sodium arsenite could be recapitulated with other oxidative stress inducers, such as hydrogen peroxide. Although the effect was relatively smaller than the effect of sodium arsenite, hydrogen peroxide also seemed to induce an antioxidant response pathway and slightly reduced LCN2 levels (Figure 3D and Figure S4C,D). Furthermore, the reduced levels of LCN2 due to sodium arsenite treatment slightly increased in the presence of N-acetyl-L-cysteine (NAC), which is known as a reactive oxygen species scavenger, although co-treatment with NAC could not completely inhibit the sodium arsenite-induced reduction in LCN2 levels (Figure 3E and Figure S4E). Therefore, the mild oxidative stress induced by sodium arsenite contributes to the reduction in LCN2 levels. However, we cannot exclude the possibility that other pathways or factors are involved in reducing LCN2 levels. Taken together, our results suggest that the induction of mild oxidative stress via sodium arsenite treatment induces the activation of the antioxidant response pathway and decreases LCN2 levels in astroglial-enriched cells at both the mRNA and protein level.

### 2.3. The Nrf2-Mediated Antioxidant Response *per se* Cannot Reduce LCN2 Levels in Astroglial-Enriched Cells

**Figure 4:**
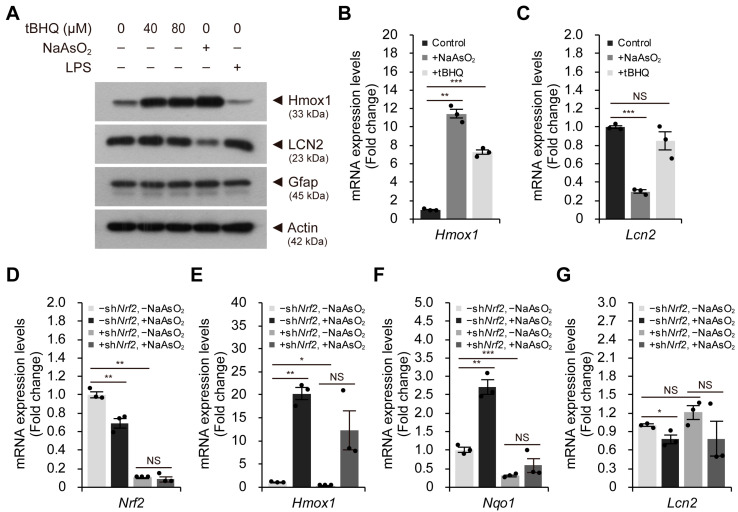
The Nrf2-mediated antioxidant response is not responsible for reduced *Lcn2* expression levels. (**A**) astroglial-enriched cells were treated with 40 or 80 μM tBHQ, 10 μM sodium arsenite (NaAsO_2_) or 100 ng/mL LPS for 1 d. The cells were then subjected to immunoblot detection of LCN2, Hmox1, and Gfap. (**B**,**C**) astroglial-enriched cells were treated with 10 μM NaAsO_2_ or 40 μM tBHQ for 1 d. The expression levels of *Lcn2* and *Hmox1* were determined by qRT-PCR, normalized against *Gapdh* levels, and expressed as the fold change relative to the control (−NaAsO_2_, −tBHQ; *n* = 3). (**D**–**G**) astroglial-enriched cells were subjected to *Nrf2* knockdown (KD) via lentiviral transduction. Negative control cells were transduced with a lentivirus harboring scrambled shRNA sequences. One day after infection, control (−sh*Nrf2*) and *Nrf2* KD (+sh*Nrf2*) cells were treated with 10 μM NaAsO_2_ or PBS for 1 d. Total RNA was isolated from the cells and subjected to qRT-PCR analysis. The expression levels of *Nrf2*, *Hmox1*, *Nqo1*, and *Lcn2* were determined by qRT-PCR, normalized against *Gapdh* levels, and expressed as the fold change relative to the control (−sh*Nrf2*, −NaAsO_2_; *n* = 3). For immunoblot analysis, β-actin was used as a loading control. Representative images of immunoblots are shown (*n* = 2). qRT-PCR data are expressed as the means ± SEM from the indicated number of samples. * *p* < 0.05, ** *p* < 0.01; *** *p* < 0.001 between two groups as indicated by the horizontal bars. NS, not significant.

To determine if activation of the Nrf2-Keap1 pathway alone is sufficient to reduce LCN2 levels, we used tertiary-butylhydroquinone (tBHQ) as an Nrf2 stabilizer without inducing oxidative stress. We investigate whether the antioxidant response pathway is directly involved in the regulation of LCN2 levels. However, tBHQ treatment did not reduce LCN2 levels in astroglial-enriched cells, despite significantly increasing Hmox1 levels at both the mRNA and protein levels (Figure 4A–C and Figure S5A,B). These results suggest that activation of the Nrf2–Keap1 pathway alone may not be sufficient to reduce LCN2 levels in astroglial-enriched cells. Thus, mild oxidative stress, induced in an Nrf2-independent manner, may be responsible for the reduced LCN2 levels in astroglial-enriched cells. 

To investigate whether the reduction in *Lcn2* expression levels by sodium arsenite can be achieved without activation of the Nrf2–Keap1 pathway, we knocked down *Nrf2* in astroglial-enriched cells. We confirmed Nrf2 knockdown by the reduced expression levels of *Nrf2* and the reduced responses of the Nrf2 target genes, *Hmox1* and *Nqo1*, to sodium arsenite (Figure 4D–F). As *Hmox1* expression is also known to be regulated via an Nrf2-independent pathway, the effect of *Nrf2* knockdown on *Hmox1* expression was less significant than its effect on *Nqo1* expression (Figure 4E,F). When we knocked down *Nrf2* in astroglial-enriched cells, reduced expression levels of *Lcn2* due to mild oxidative stress were still observed (Figure 4G). Therefore, our data suggest that reduced LCN2 levels in response to sodium arsenite treatment in astroglial-enriched cells were not simply mediated by activation of the Nrf2–Keap1 pathway. Although further detailed investigation may be required, a method to reduce LCN2 levels in astroglial-enriched cells will provide insights into how to reduce reactive-astrocyte-induced neurotoxicity in various neurological disorders.

## 3. Discussion

To investigate the regulatory mechanisms of LCN2 in the central nervous system, we cultured primary glial cells isolated from the cortical regions of mouse brains. According to previous studies, cultured cortical cells are composed of mixed populations of glial cells, including astrocytes and microglia [25,26,27]. Based on the suggestion from a previous report, here, we refer to these cells as astroglial-enriched cells [25]. In this study, we treated these cells with sodium arsenite, which is generally regarded as a cytotoxic reagent that induces the production of reactive oxygen species, DNA damage, autophagosome formation, and even apoptosis [24,28,29]. However, sodium arsenite treatment at a concentration of 10 μM did not compromise the viability of astroglial-enriched cells. 

Previous studies have shown that *Lcn2* expression is upregulated by the activation of NF-κB signaling, which is essential for the production of various inflammatory cytokines and for cell growth and survival [19,20]. Interestingly, intracellular crosstalk between NF-κB signaling and the Nrf2–Keap1 pathway has previously been reported [23]. Briefly, increased levels of Hmox1 induced by activation of the Nrf2–Keap1 pathway block the activation of inhibitory κB kinase (IKK), resulting in the stabilization of IκB and suppression of NF-κB signaling. Therefore, we first speculated that crosstalk between the Nrf2–Keap1 pathway and NF-κB signaling may be required for the mild oxidative stress-induced reduction in LCN2 levels. However, increased levels of Hmox1 did not seem to affect IκB levels in astroglial-enriched cells (see Figure 2C). Thus, reduced LCN2 levels under mild oxidative stress may not result from the suppression of NF-κB signaling. In fact, reduced *Lcn2* expression can also be observed without LPS stimulation or activation of the NF-κB signaling pathway. 

We have recently reported that under neuroinflammatory conditions induced by LPS treatment, LCN2 production can be reduced by bortezomib treatment and autophagic degradation of LCN2 can be accelerated by torin 1 treatment [30]. Proteasome inhibition through bortezomib treatment suppresses NF-κB signaling due to the stabilization of IκB even under neuroinflammatory conditions, in contrast to sodium arsenite treatment. In reactive astrocytes, LCN2 is a short-lived protein with a half-life of ~30 min and is targeted to the autophagy–lysosome pathway for degradation. However, when secreted from the cells, it induces the loss of nearby neurons. We have clearly demonstrated that reduced secretion of LCN2 from reactive astrocytes has beneficial effects on neurons, leading to the restoration of neuronal viability under neuroinflammatory conditions. 

In this study, we demonstrated that LCN2 levels can be reduced by mild oxidative stress in an Nrf2-independent manner, regardless of neuroinflammatory conditions. Mild oxidative stress may represent a novel strategy for reducing LCN2 levels in astroglial-enriched cells, thereby preventing its potentially neurotoxic effects without altering or impacting the NF-κB signaling pathway. Therefore, pharmaceutical approaches targeting LCN2 in neurological diseases have emerged and regulating LCN2 levels may become an important therapeutic approach for various neurological disorders.

## 4. Materials and Methods

### 4.1. Mouse Experiments

Wild-type CD-1 (ICR) mice were purchased from Raon Bio (Yongin, Republic of Korea) and were transferred to the mouse facility before the experiments. All mice were housed in plastic cages with ad libitum access to food and water under a 12 h light/dark cycle. All mouse experiments, including the isolation of primary cells from postnatal mouse brains, were approved by the University of Seoul Institutional Animal Care and Use Committee (approval no. UOS-IACUC-2020-03-A, UOS IACUC-2021-01-TA). All animal procedures were performed in accordance with relevant guidelines and regulations approved by the UOS IACUC.

### 4.2. Primary Astroglial-Enriched Cell Culture

Astrocytes were enriched and subsequent processes were performed as previously described [26]. Briefly, brains were isolated from pups on postnatal day 1 and placed in sterilized Petri dishes containing Hank’s balanced salt solution (HBSS). The isolated brains were further processed by removal of the cerebellum, olfactory bulbs, and meninges to obtain the cortical regions. After cutting the cortex into small pieces (1–2 mm^3^), the collected pieces (from 3 to 4 pups) were incubated in a 0.05% trypsin/EDTA solution (CellGro, Manassas, VA, USA) for 30 min at 37 °C. To prevent over-trypsinization, an equal volume of cell culture medium (Dulbecco’s modified Eagle medium [DMEM] supplemented with 10% fetal bovine serum, 20 mM L-glutamine, and 1% antibiotics/antimycotics) was added and the samples were gently mixed. After centrifugation at 300× *g* for 5 min, the supernatant was removed and the pelleted cortical pieces were gently triturated in 5 mL of additional cell culture medium. After adjusting the volume of the cell suspension to 10 mL with additional cell culture medium, the cell suspension (from 3 to 4 pups) was transferred into a previously prepared 100 mm cell culture dish coated with poly-D-lysine (MW 30,000–70,000; Sigma-Aldrich, St. Louis, MO, USA). The medium was changed every 2 days. After 7–8 days, confluent astroglial-enriched cells were split in half and passaged. After 12–14 days, the cells were seeded on cell culture dishes or cover glasses, depending on the experiment, 48 h before beginning the experiment.

### 4.3. Immunoblot Analysis

Cell lysates were prepared from astroglial-enriched cells treated with sodium arsenite, tertiary-butylhydroquinone (tBHQ), lipopolysaccharide (LPS), or other chemicals, using radioimmunoprecipitation (RIPA) buffer. After lysis for 30 min on ice, the samples were centrifuged at 13,000× *g* for 15 min at 4 °C, and the resulting supernatant was collected. The protein concentration of each sample was determined using the Pierce^TM^ BCA assay kit (Thermo Fisher Scientific, Waltham, MA, USA). Proteins (15–30 μg) were separated via sodium dodecyl sulfate (SDS) polyacrylamide gel (10%) electrophoresis at 25 mA (per gel) for 60–75 min. After electrophoresis, the separated proteins were transferred to polyvinylidene fluoride (PVDF) membranes at 100 V for 90 min. The transferred membranes were blocked with 5% skim milk in TBST (Tris-buffered saline [TBS] containing 0.05% Tween-20) for 1 h at room temperature and incubated with primary antibodies suspended in 5% skim milk/TBST at 4 °C overnight. After primary antibody incubation, the membranes were washed with TBST and subsequently incubated with secondary antibodies (horseradish-peroxidase [HRP]-conjugated goat anti-mouse IgG or rabbit IgG) suspended in 5% skim milk/TBST for 1 h at room temperature. Finally, chemiluminescent signals were detected and images were captured using an enhanced chemiluminescence solution (WSE-7120L; ATTO, Tokyo, Japan) with a ChemiDoc system (Bio-Rad, Hercules, CA, USA). The antibodies used in this study are as follows: anti-LCN2 (PA5-79590; rabbit polyclonal, 1:1000; Thermo Fisher Scientific); anti-Gfap (MAB360; mouse monoclonal, 1:1000; Millipore, Burlington, MA, USA); anti-Hmox1 (5061; rabbit polyclonal, 1:1000; Cell Signaling Technology, Danvers, MA, USA); anti-Nqo1 (ab34173; rabbit polyclonal, 1:2000; Abcam, Cambridge, UK); anti-NF-κB/p65 (51-0500; rabbit polyclonal, 1:1000; Thermo Fisher Scientific); anti-IκBα (MA5-15132; mouse monoclonal, 1:1000; Thermo Fisher Scientific); anti-β-actin (SC-47778; mouse monoclonal, 1:1000; Santa Cruz Biotechnology, Dallas, TX, USA); HRP-conjugated goat anti-mouse or anti-rabbit IgG (ADI-SAB-100-J or ADI-SAB-300-J; goat polyclonal, 1:10,000; Enzo Life Sciences, Farmingdale, NY, USA).

### 4.4. Quantitative Reverse Transcription Polymerase Chain Reaction (qRT-PCR) Analysis

After chemical treatment or lentivirus-mediated cell transduction, total RNA was isolated from cells using TRI Reagent (Molecular Research Center, Cincinnati, OH, USA) following the manufacturer’s protocol and reconstituted in RNase-free ddH_2_O. RNA concentration was measured using a NanoDrop One^TM^ instrument (Thermo Fisher Scientific), and 1 μg of RNA was incubated with DNase I (Invitrogen, Carlsbad, CA, USA) for 15 min at room temperature. After inactivating DNase I, following the manufacturer’s protocol, the prepared RNA samples were used as templates for reverse transcription using an oligo(dT) primer (18-mer) and SuperiorScript II reverse transcriptase (Enzynomics, Daejeon, Republic of Korea) following the manufacturer’s protocol. cDNA samples generated from mRNA were used as templates for qRT-PCR using 2× SYBR Master Mix (Enzynomics), primer pairs, and an iCycler system (IQ5; Bio-Rad). The mRNA expression levels of *Lcn2*, *Nrf2*, *Hmox1*, and *Nqo1* were normalized to the levels of glyceraldehyde 3-phosphate dehydrogenase (*Gapdh*). The primers used in this study were as follows: *Lcn2*-F, 5′-CTG AAT GGG TGG TGA GTG TG-3′; *Lcn2*-R, 5′-GCT CTC TGG CAA CAG GAA AG-3′; *Nrf2*-F, 5′-ATC CAG ACA GAC ACC AGT GGA TC-3′; *Nrf2*-R, 5′-GGC AGT GAA GAC TGA ACT TTC A-3′; *Hmox1*-F, 5′-CCT GGT GCA AGA TAC TGC CC-3′; *Hmox1*-R, 5′-GAA GCT GAG AGT GAG GAC CCA-3′; *Nqo1*-F, 5′-GGT AGC GGC TCC ATG TAC TC-3′; *Nqo1*-R, 5′-CAT CCT TCC AGG ATC TGC AT-3′; *Tnf*-F, 5′-TCT CAT CAG TTC TAT GGC CC-3′; *Tnf*-R, 5′-GGG AGT AGA CAA GGT ACA AC-3′; *Il1a*-F, 5′-CAC CAA AGA ACA AAG TCG GG-3′; *Il1a*-R, 5′-GGA AGG TTC CTG TAC ATG GTA C-3′; *Gapdh*-F, 5′-GGC ATT GCT CTC AAT GAC AA-3′; and *Gapdh*-R, 5′-CTT GCT CAG TGT CCT TGC TG-3′.

### 4.5. MTT Assay

Astroglial-enriched cells were seeded into 24-well cell culture plates and treated with various concentrations of sodium arsenite. After 24 h of incubation, the medium was replaced with a thiazolyl blue tetrazolium bromide (MTT) solution (500 μg/mL of MTT in DMEM) and the cells were incubated for 3 h at 37 °C. The converted MTT was solubilized with dimethyl sulfoxide (DMSO) and measured using a microplate reader at a wavelength of 570 nm with background subtraction at 650 nm.

### 4.6. Immunofluorescence Analysis

Astroglial-enriched cells were seeded on poly-D-lysine-coated coverslips 2 days before beginning the experiments and treated with sodium arsenite overnight. Cells were washed briefly with phosphate-buffered saline (PBS), fixed with 4% paraformaldehyde/PBS for 10 min at room temperature, permeabilized with 0.3% Triton X-100/PBS for 10 min at room temperature, and blocked with 3% bovine serum albumin/PBS for 1 h at room temperature. The cells were then incubated with primary antibodies (1:500, anti-LCN2 and anti-Gfap) at 4 °C overnight. After primary antibody incubation, the cells were washed with PBS and incubated with fluorophore-conjugated secondary antibodies (1:1000, Alexa Fluor 488-conjugated goat anti-mouse IgG and 555-conjugated goat anti-rabbit IgG) and 4, 6-diamidino-2-phenylindole (DAPI) for 1 h at room temperature. The cells were washed with PBS and mounted using the Prolong Gold Antifade Reagent (Thermo Fischer Scientific).

### 4.7. Lentivirus Production

Lentivirus production in Lenti-X HEK293T cells (Takara Bio, Kusatsu, Japan) was performed using the general calcium phosphate transfection method, as previously described [31]. pLKO.1-scrambled was purchased from Addgene and pLKO.1-sh*Nrf2* was generated using the oligonucleotide targeting mouse *Nrf2* [23]. Briefly, one day before transfection, HEK293T cells were split and seeded on poly-D-lysine-coated 100 mm cell culture dishes at 2.0 × 10^6^ cells/dish. Three hours before transfection, the medium was changed to achieve optimal transfection conditions. The concentrations of all plasmids (pLKO.1-scrambled shRNA, pLKO.1-sh*Nrf2*, psPAX2, and pMD2.G) were measured using a NanoDrop One^TM^ instrument. Ten μg of pLKO.1-sh*Nrf2* (or pLKO.1-scrambled shRNA as a negative control), 8 μg of psPAX2, and 3 μg of pMD2.G were mixed with 500 μL of HEPES-buffered saline (HEBS) and 250 μL of CaCl_2_, and the volume of the mixture was adjusted with ddH_2_O to 1 mL. After incubation for 30 min at room temperature, the mixture was sprinkled evenly onto the prepared cells and mixed with the medium by gentle shaking. After an overnight incubation, the medium was replaced with fresh medium, incubated for an additional 24 h, and then collected. The collected medium (containing lentivirus) was filtered through a 0.45 μm low-protein-binding filter (Pall Corporation, Port Washington, NY, USA), mixed with 3 mL of Lenti-X™ concentrator (Takara Bio), and incubated at 4 °C overnight. The mixture was centrifuged at 1500× *g* for 45 min at 4 °C, and the pelleted lentivirus was resuspended in PBS and stored at −80 °C before use. Before infection, the lentiviral titer was measured using a qPCR Lentivirus Titer Kit (Applied Biological Materials, Richmond, BC, Canada), and the multiplicity of infection (MOI) was calculated based on the titer. Astroglial-enriched cells were transduced with lentiviral vectors at an MOI of 10 in the presence of 4 μg/μL polybrene. The shRNA sequences used in this study were as follows: scrambled-sense, 5′-CCT AAG GTT AAG TCG CCC TCG CTC GAG CGA GGG CGA CTT AAC CTT AGG-3′ and sh*Nrf2*-sense, 5′-CCA AAG CTA GTA TAG CAA TAA CTC GAG TTA TTG CTA TAC TAG CTT TGG-3′.

### 4.8. Statistical Analysis

Two-tailed unpaired Student’s *t*-tests were used to compare data between the two groups. Differences were considered statistically significant at *p* < 0.05.

## Data Availability

Not applicable.

## Supplementary Materials

The supporting information can be downloaded at: https://www.mdpi.com/article/10.3390/ijms242115864/s1.
