# Supplementary material for: Mild Oxidative Stress Induced by Sodium Arsenite Reduces Lipocalin-2 Expression Levels in Cortical Glial Cells
Source: Int J Mol Sci. 2023 Nov 1;24(21):15864. doi: 10.3390/ijms242115864 (PMC10649205; doi:10.3390/ijms242115864)
Supplement: Supplementary file 1 [file ijms-24-15864-s001.zip › ijms-2675487-supplementary.pdf]

Supplemental Figures

Mild Oxidative Stress Induced by Sodium Arsenite Reduces Lipocalin-2 Expression Levels in Cortical Glial Cells

Ye-Jin Cho, So-Hyun Park and Kwon-Yul Ryu *

Department of Life Science, University of Seoul, Seoul 02504, Republic of Korea; fifi1022@naver.com (Y.-J.C.); sohyun1231@naver.com (S.-H.P.)

* Correspondence: kyryu@uos.ac.kr

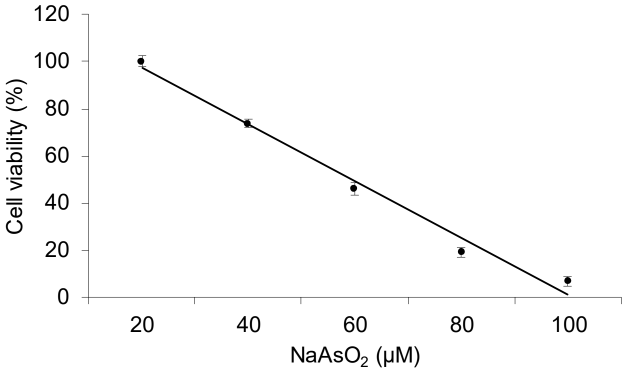

Figure S1. Determination of LC50 of sodium arsenite ($NaAsO_2$) in astroglial-enriched cells. Astroglial-enriched cells were treated with $NaAsO_2$ at the indicated concentrations up to 100 µM for 1 d. The viability of cells was measured using the MTT assay (n=3). Cell viability was expressed as a percentage relative to the control in which no reduced viability was observed (20 µM $NaAsO_2$).

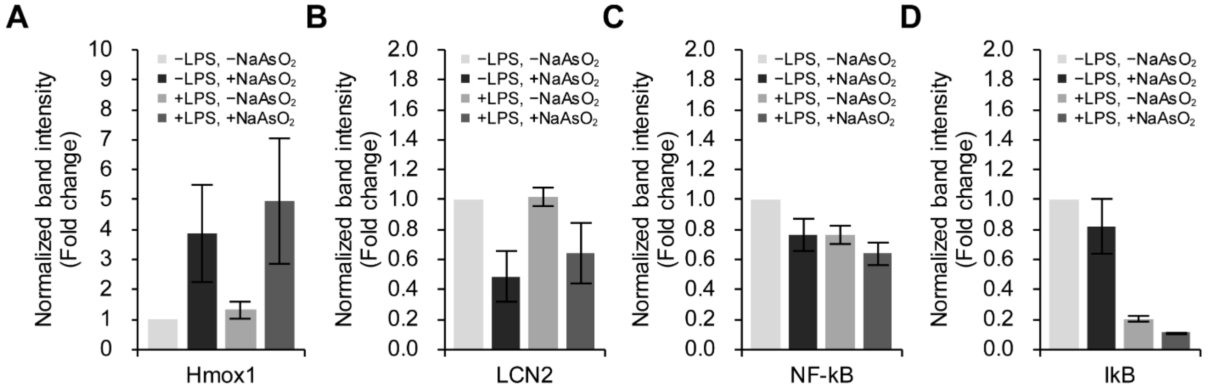

Figure S2. Quantification of immunoblot bands. (**A,B**) Band intensities of Hmox1 and LCN2 in Figure 2B were normalized to the loading control β-actin and expressed as the fold change relative to the control (−LPS, −$NaAsO_2$; n=3). (**C,D**) Band intensities of NF-κB and IκB in Figure 2C were normalized to the loading control β-actin and expressed as the fold change relative to the control (−LPS, −$NaAsO_2$; n=3). All bands intensities were quantified using ImageJ software (version 1.8.0).

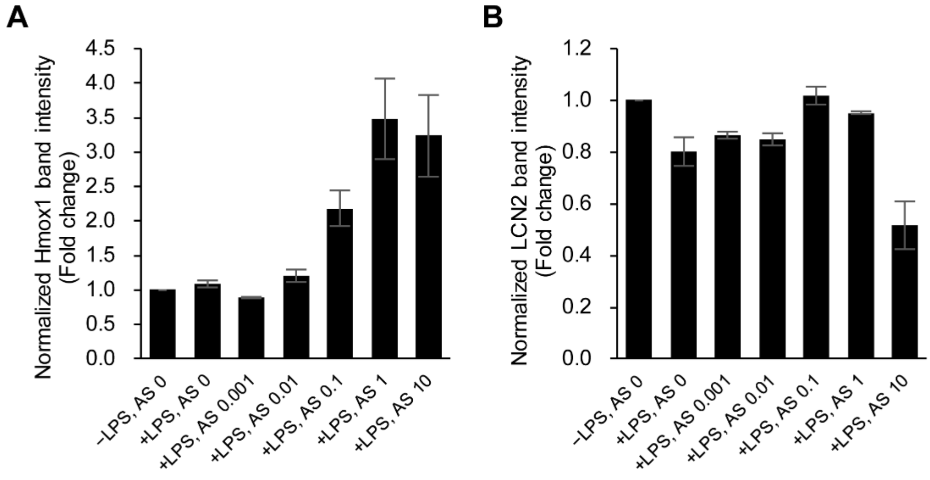

Figure S3. Quantification of immunoblot bands. (**A,B**) Band intensities of Hmox1 and LCN2 in Figure 3B were normalized to the loading control β-actin and expressed as the fold change relative to the control (−LPS, −$NaAsO_2$; n=2). All bands intensities were quantified using ImageJ software (version 1.8.0). The numbers (0, 1, 10, 50, and etc) indicate the concentration (μM) of chemicals. AS, $NaAsO_2$.

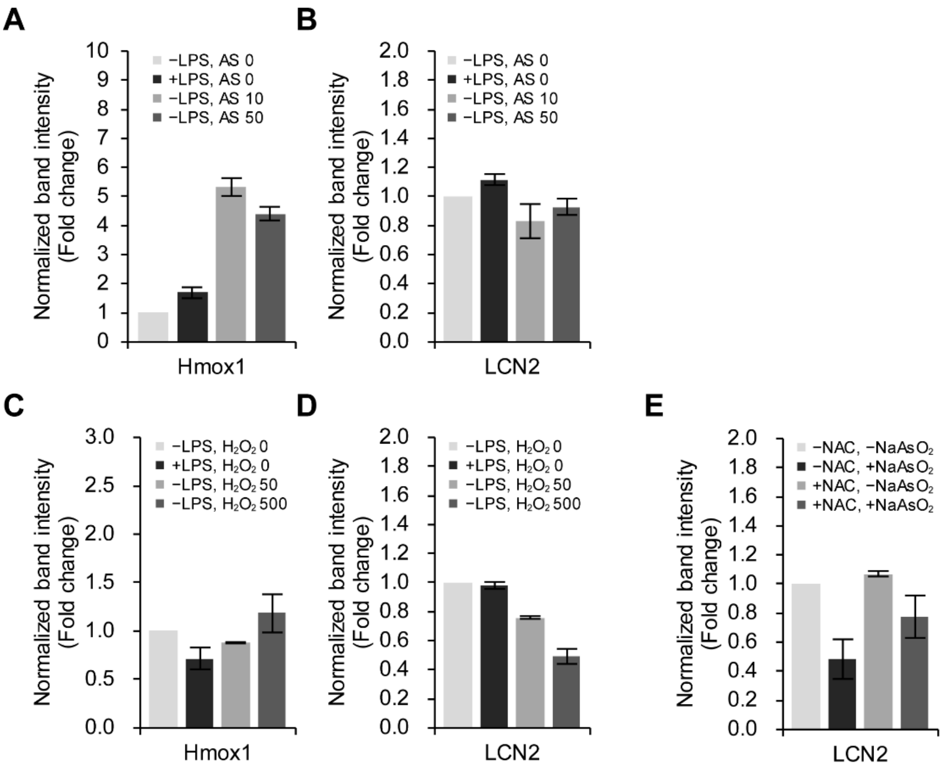

Figure S4. Quantification of immunoblot bands. (**A,B**) Band intensities of Hmox1 and LCN2 in Figure 3C were normalized to the loading control β-actin and expressed as the fold change relative to the control (−LPS, −$NaAsO_2$; n=2). (**C,D**) Band intensities of Hmox1 and LCN2 in Figure 3D were normalized to the loading control β-actin and expressed as the fold change relative to the control (−LPS, − H_2O_2; n=2). (**E**) Band intensities of LCN2 in Figure 3E were normalized to the loading control β-actin and expressed as the fold change relative to the control (−NAC, −$NaAsO_2$; n=2). All bands intensities were quantified using ImageJ software (version 1.8.0). The numbers (0, 1, 10, 50, and etc) indicate the concentration (μM) of chemicals. AS, $NaAsO_2$, NAC, N-acetyl-L-cysteine.

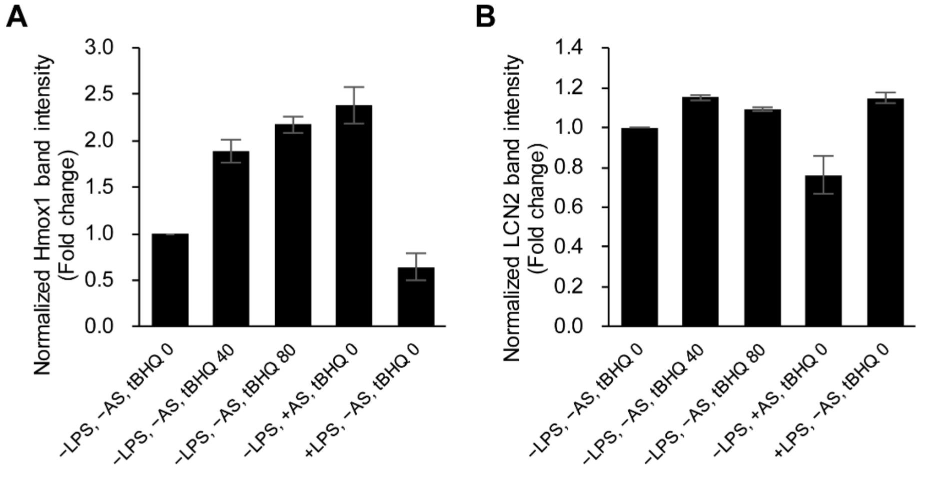

Figure S5. Quantification of immunoblot bands. (**A,B**) Band intensities of Hmox1 and LCN2 in Figure 4A were normalized to the loading control β-actin and expressed as the fold change relative to the control (−LPS, −$NaAsO_2$, −tBHQ; n=2). All bands intensities were quantified using ImageJ software (version 1.8.0). The numbers (0, 40, and 80) indicate the concentration (μM) of tBHQ. AS, $NaAsO_2$.
